# ACT001, a novel PAI-1 inhibitor, exerts synergistic effects in combination with cisplatin by inhibiting PI3K/AKT pathway in glioma

**DOI:** 10.1038/s41419-019-1986-2

**Published:** 2019-10-07

**Authors:** Xiaonan Xi, Ning Liu, Qianqian Wang, Yahui Chu, Zheng Yin, Yahui Ding, Yaxin Lu

**Affiliations:** 1 College of Pharmacy, Nankai University, 300350 Tianjin, People’s Republic of China; 2 State Key Laboratory of Medicinal Chemical Biology, Nankai University, 300350 Tianjin, People’s Republic of China

**Keywords:** Cancer therapy, CNS cancer

## Abstract

PAI-1 plays significant roles in cancer occurrence, relapse and multidrug resistance and is highly expressed in tumours. ACT001, which is currently in phase I clinical trials for the treatment of glioblastoma (GBM). However, the detailed molecular mechanism of ACT001 is still unclear. In this study, we investigated the effects of ACT001 on glioma cell proliferation and clarified its mechanism. We discovered that PAI-1 was the direct target of ACT001 by a cellular thermal shift assay. Then, the interaction between ACT001 and PAI-1 was verified by Biacore assays, thermal stability assays and ACT001 probe assays. Furthermore, from the proteomic analysis, we found that ACT001 directly binds PAI-1 to inhibit the PI3K/AKT pathway, which induces the inhibition of glioma cell proliferation, invasion and migration. Moreover, the combination of ACT001 and cisplatin showed a synergistic effect on the inhibition of glioma in vitro and in vivo. In conclusion, our findings demonstrate that PAI-1 is a new target of ACT001, the inhibition of PAI-1 induces glioma inhibition, and ACT001 has a synergistic effect with cisplatin through the inhibition of the PAI-1/PI3K/AKT pathway.

## Introduction

Glioblastoma (GBM) is the most common and most aggressive primary malignant brain tumour in adults, with a high morbidity, high mortality and poor patient prognosis^1^. Standard-of-care therapies include surgical resection followed by postoperative radiotherapy and chemotherapy^2^. However, GBM patients still have a poor median survival rate due to resistance to chemoradiotherapy. Platinum-based drugs, including cisplatin, are commonly used in tumour therapy^3,4^. Cisplatin has been used as a neoadjuvant therapy with temozolomide and as an adjuvant therapy with carmustine in malignant glioma therapy^5,6^. However, drug resistance and serious side effects are some of the inadequacies of platinum-based drugs for the treatment of GBM^7–9^. The treatment of glioma urgently needs the development of new therapeutic drugs or establishment of new combination therapies to improve patient survival and reduce the side effects of conventional chemotherapy. Therefore, new therapeutic drugs and new drug targets need to be developed.

ACT001 (also known as dimethylaminomicheliolide, i.e., DMAMCL), a guaianolide sesquiterpene lactone developed by Accendatech Co., Ltd. (Tianjin, China) was certified as an orphan drug by the Food and Drug Administration (FDA) in the United States and by the European Union and is currently undergoing phase II clinical trials^10,11^. ACT001 has shown excellent results in controlling the growth of GBM in phase I clinical trials^12^. However, the mechanism of ACT001 is not clear, which restricts the clinical application of ACT001 and limits the selection of patients for clinical experiments. In a previous study, we found that ACT001 selectively activated PKM2 through covalent binding at residue cysteine 424 to promote tetramer formation, which inhibited tumour metabolism^12^. ACT001, a fumarate salt form of micheliolide (MCL), converts to MCL consistently under physiological conditions^10,12^.

Plasminogen activator inhibitor-1 (PAI-1) is a protease inhibitor and is related to poor clinical prognosis^13–15^. PAI-1 participates in tissue remodelling by blocking the binding of integrin α_v_β_3_ to the vitronectin protein in the extracellular matrix^16^. PAI-1 regulates tumour growth through angiogenesis and is involved in the migration, invasion and adhesion of cancer cells^17–19^. PAI-1 is also associated with multi-drug resistance^13,20^. A high expression of PAI-1 leads to drug resistance, which indicates that targeting PAI-1 is an important strategy for the treatment of GBM. In addition, studies have confirmed that GBM contains a large amount of PAI-1, and this protein is related to the metastasis of the tumour^21^.

Here, in this article, we report that PAI-1 is overexpressed in glioma tissues and cell lines. ACT001 targets PAI-1 and inhibits glioma cell proliferation, invasion and metastasis through the PAI-1/PI3K/AKT pathway. Moreover, we demonstrate that ACT001 can enhance the antitumour effects of cisplatin in vitro and in vivo.

## Materials and methods

### Materials

U118MG cells were purchased from the National Infrastructure of Cell Line Resource (China). Recombinant human PAI-1 was purchased from USA Immuno Clone Biosciences. ACT001 was provided by Accendatech Co., Ltd. (Tianjin, China). Biotion-S-MCL (inactive) and biotin-MCL (active) were synthesized as previously described^12^. MCL was obtained according to a previously described method^10^.

### Cell culture and transfection of siRNAs

U118MG cells were cultured in Dulbecco’s modified Eagle’s medium (Gibco) supplemented with 10% foetal bovine serum (Gibco), 100 IU/mL penicillin and 0.1 mg/mL streptomycin (HyClone) in a humidified incubator containing 5% CO_2_ at 37 °C.

PAI-1 siRNA and the negative control siRNA were purchased from Santa Cruz Biotechnology, Inc. The cells were plated in 6-well plates (Corning, America) at a concentration of 3 × 10^5^ cells per plate and grown overnight before transfection. Transfection was performed using Lipofectamine 2000 reagent (Invitrogen, Carlsbad, USA) in accordance with the manufacturer’s protocol. The cells were collected for western blot analysis and sample preparation for mass spectrometry (MS) analysis after being transfected for 48 h or 72 h. After being transfected for 24 h, 10 μM ACT001 or blank solvent was added for the wounding healing assay, cell invasion assay, apoptosis assay and tube formation assay.

### Cellular thermal shift assay

The cellular thermal shift assay (CETSA) is based on the biophysical principle of ligand-induced thermal stabilization of the target proteins. The energetic coupling of the ligand-binding and receptor-melting reactions results in this stability phenomenon^22,23^. For thermal proteome profiling (TPP), we used the protocol of Savitski et al. with some modifications^24^: the cell extract was divided into two parts. A solution of the compound in phosphate-buffered saline (PBS)/dimethyl sulfoxide (DMSO) or PBS/DMSO alone as the vehicle (ACT001: 400 μM; MCL: 400 μM) was added to the cell extract to a final 1% PBS/DMSO concentration. Then, the two groups were incubated for 40 min at 37 °C. After incubation, the two groups were divided into four parts and placed in 0.2-mL PCR tubes, each of which contained 100 μL. One sample contained the compound, and one sample contained the vehicle; both were heated in parallel for 3 min to the set temperature (the temperatures were 41, 45, 49, and 53 °C), followed by a 3-min incubation time at room temperature. Subsequently, the extract was centrifuged at 12,000×*g* for 20 min at 4 °C. The supernatant was collected; the concentration of proteins was determined by the bicinchonininc acid (BCA) method, and then the proteins were fractionated by SDS gel electrophoresis and subjected to sample preparation for MS analysis or western blot analysis. For the isothermal dose-response (ITDR) experiments, MCL was tested as a six-point serial dilution (800 μM, 400 μM, 100 μM, 10 μM,1 μM, 0 μM) at 56 °C following the above steps for the experiment with the extract.

### Surface plasmon resonance assay

The Biacore 3000 analytical systems were from GE Healthcare Life Sciences (Boston, America). The CM5 sensor chip was from Xantec (Duesseldorf, Germany). Because of refractive index variations, an empty flow cell was used to correct all recorded sensor grams. PAI-1 was immobilized at 50 μg/mL in pH 4.5 sodium acetate buffer, resulting in 9000 immobilized resonance units. In the ACT001 surface plasmon resonance (SPR) experiment, 1× PBS (pH 7.4) was used. In the MCL and PAI-039 SPR experiments, 1× PBS containing 1% DMSO (pH 7.4) was used. All SPR experiments were carried out with a contact time of 60 s and a dissociation time of 60 s at a flow rate of 30 μL/min. The binding of ACT001 to immobilized PAI-1 was monitored by applying ACT001 (100 μM–1000 μM) in 1× PBS (pH 7.4). The binding of MCL and PAI-039 to immobilized PAI-1 was monitored by applying either MCL (7.8 μM–125 μM) or PAI-039 (390 nM–18.75 μM) in 1× PBS (pH 7.4) containing 1% DMSO. After being incubated with different concentrations of ACT001 (0.78125 μM–50 μM) in 1× PBS (pH 7.4) at 37 °C overnight, the solution was injected into the PAI-1 bounded sensor chip. The equilibrium dissociation constant (*K*_D_) was obtained using BIA evaluation software.

### Pull-down assay

The pull-down experiments were carried out according to the protocol of Li et al.^12^. U118 cells were harvested and lysed in RIPA buffer (Solarbio, Beijing, China) containing a mix of protease inhibitors (Solarbio, Beijing, China). Following a 10-min incubation on ice, the cells were sonicated and then centrifuged at 14,000×*g* for 20 min at 4 °C. The supernatant was collected, and the protein content was determined with the BCA method. The supernatant was then divided into two parts, each containing 1 mg of protein. The two samples were either incubated with 20 µM biotin-MCL or biotin-S-MCL in RIPA buffer overnight at 4 °C. The two samples were mixed with an excess of pre-cooled methanol, precipitated at −80 °C for 30 min and centrifuged at 14,000×*g* for 20 min at 4 °C. PBS (containing a protease inhibitor cocktail and 0.1% SDS) was then used to dissolve the precipitated proteins. Next, 30 µL of streptavidin beads were pre-washed, added to each sample and incubated for 1 h at room temperature. Then, the streptavidin beads were washed three times with RIPA buffer, and the bead-bound proteins were eluted, separated by SDS-PAGE, and underwent western blot experiments.

### Molecular docking

The protein structure of PAI-1 was downloaded from the PDB database (http://www.rcsb.org). The Schrodinger software was selected to optimize the obtained protein structure. Then, we assigned bond orders, added hydrogens, and found overlaps. Finally, we determined the minimization energy of the PAI-1 protein. The structures of ACT001, MCL and PAI-039 were built using ChemDraw. Then, the prepared ACT001, MCL and PAI-039 were analysed with Schrodinger (LigPred module). Glide ligand docking was employed to dock ACT001, MCL and PAI-039 into the active site of PAI-1. Glide XP (extra precision) mode was used for all docking calculations.

### Western blot assay

Total protein was collected using RIPA buffer containing a mix of protease inhibitors and quantified with a BCA protein assay kit. Cell lysates (30 μg or 50 μg per sample) were resolved by SDS-PAGE, and then the proteins were transferred to PVDF membranes. After being incubated in blocking buffer, the PVDF membranes were incubated with primary antibodies against PI3K (1:1000, Affinity, AF6242), p-PI3K (1:1000, Affinity, AF3242), AKT (1:1000, Affinity, AF6261), p-AKT (1:1000, Affinity, AF0016), GAPDH (1:7500, Proteintech), Snail (1:1000, CST), vimentin (1:1000, CST), β-catenin (1:1000, CST), E-cadherin (1:1000, CST) and PAI-1 (1:1000, CST) at 4 °C overnight. A secondary goat anti-rabbit HRP-IgG (Proteintech) was used to detect the primary antibodies, and the chemiluminescence intensities were detected using the ECL system (Millipore, Billerica, USA).

### MTT assay

Cell viability was measured by MTT assay. In total, 3 × 10^3^ cells were plated in 96-well culture plates. After 24 h, cells were treated with drugs for 72 h and subsequently incubated with 20 μL of MTT (5 mg/mL) at 37 °C for 4 h. The medium was then removed, and 100 μL of DMSO was added to dissolve the formazan. The absorbance was measured at 570 nm using a microplate reader (MD, Austria). Each experiment was repeated at least three times.

### Drug synergy assessment

Cell viability was measured using the MTT assay. The cells were exposed to different concentrations of ACT001 (1.875, 3.75, 7.5, 15, 30 and 60 μM), cisplatin (1.875, 3.75, 7.5, 15, 30 and 60 μM) and combinations of both drugs for 72 h. Synergy was assessed using the Bliss independence model as described in the literature^25^.

### Cell survival crystal violet staining

Cells that were exposed to ACT001, cisplatin and a combination of the two drugs for 72 h were fixed with precooled methanol and subsequently incubated with crystal violet for 30 min. Then, the cells were washed with 1× PBS, and photos were taken using a microscope (Nikon, Japan).

### Wound healing assays and invasion assays

Cell migration was detected and evaluated by wounding healing (scratch) assays. Briefly, the cells were plated in 6-well plates (3 × 10^5^ cells/plate) and incubated to generate confluent cultures. Scrapes were made with a 200-μL sterile pipette tip, and the cells were washed with PBS buffer and then treated with ACT001 (7.5 μM), cisplatin (7.5 μM) or a combination of the two drugs for 12 h. For the ACT001 (10 μM)/siRNA PAI-1 group, after the 24 h siRNA PAI-1 transfection, scrapes were made, and the cells were treated with or without ACT001 for another 48 h. Finally, photos of cell migration were taken with a microscope.

Invasion assays were carried out using 24-well plates (Corning, America) and 8 μm transwell chambers (Corning, America). Matrigel (Matrigel:medium, 1:1) was added to the top chamber and incubated at 37 °C for ~30 min. After the Matrigel solidified, 50,000 cells in 200 μL of serum-free medium containing ACT001 (7.5 μM), cisplatin (7.5 μM) or a combination of the two drugs were added to the top chamber. For the ACT001 (10 μM)/siRNA PAI-1 group, after the 24 h siRNA PAI-1 transfection, the cells were collected and suspended in serum-free medium (2.5 × 10^5^/mL) with or without ACT001. Then, 200 μL of cells were added to the top well. The lower chambers were filled with 600 μL of medium with 10% FBS. After being incubated in an incubator for 24 h, the filter membranes in the chambers were removed and washed with PBS three times to remove the medium and Matrigel. Then, the cells that crossed to the underside of the filter membrane were fixed with cold methanol for 30 min, stained with 0.1% crystal violet, and counted under a microscope.

### Apoptosis assays

Apoptosis was determined with an annexin V-FITC/PI apoptosis detection kit (Solarbio Life Sciences, Beijing, China) with a BD LSR Fortessa flow cytometer (BD Biosciences, San Jose, CA, USA). After treatment with ACT001 (7.5 μM), cisplatin (7.5 μM) or a combination of the two drugs for 48 h, the cells were collected (1 × 10^6^ per sample). For the ACT001 (10 μM)/siRNA PAI-1 group, after the 24 h siRNA PAI-1 transfection, the cells were treated with or without ACT001 for another 48 h. The cells were washed with cold PBS three times. Then, the cells were suspended in 1× binding buffer, followed by centrifugation at 300×*g* for 10 min. Subsequently, the cells were re-suspended in 1 mL of 1× binding buffer. A 100-μL cell suspension was transferred to a new tube, and 5 μL of annexin V-FITC was added. After being incubated in the dark at room temperature for 10 min, 5 μL of PI was added, and the sample was incubated in the dark at room temperature for 5 min. Finally, 400 μL of PBS was added. All samples were analysed with a FACSCalibur flow cytometer, and data were processed with FlowJo 7.6.1 software.

### Phalloidin staining assays

After exposure to ACT001 (7.5 μM), cisplatin (7.5 μM) or a combination of the two drugs for 72 h, the cells grown on cover slips were fixed with cold methanol for 30 min, stained with phalloidin and DAPI and then imaged with a microscope.

### Vasculogenic mimicry assays

Vasculogenic mimicry (VM) assays were carried out using 96-well plates (Corning, America). Matrigel (Matrigel:serum-free medium, 1:3; 75 μL/well) was added to a 96-well plate and incubated at 37 °C for ~30 min until the Matrigel solidified. After the 24 h siRNA PAI-1 transfection, 20,000 cells suspended in 200 μL of medium with or without ACT001 (10 μM) were added to the 96-well plate. After being cultured in an incubator for ~4 h, photos were taken with a microscope.

### Preparation and analysis of the proteomic samples

After separating the proteins by SDS-PAGE and staining the gel using a reversible Coomassie stain, the gel lanes were cut into appropriate slices (~1 mm^3^/slice); the gel slices were then transferred into microcentrifuge tubes. A total of 500 μL of 50 mM ammonium bicarbonate/50% ACN was added to the gel slices, and the sample was incubated at 37 °C to destain the gel slices. The destaining buffer was removed, and the process was repeated until the stain disappeared. A total of 200 μL of 10 mM DTT (prepared with 25 mM ammonium bicarbonate solution) was added, and the samples were incubated at 56 °C for 1 h. After the DTT solution was removed from the gel slices, 55 mM IAA solution (prepared with 25 mM ammonium bicarbonate solution) was added, and the samples were incubated in the dark for 45 min. After the IAA solution was removed, the gel slices were rinsed with 25 mM ammonium bicarbonate three times, followed by 50 mM ammonium bicarbonate/50% ACN three times. Then, 200 μL of ACN was added to shrink the gel pieces. The ACN was removed, and the gel pieces were allowed to air dry to remove residual ACN. An appropriate volume of 0.01 mg/mL trypsin solution (prepared with 25 mM ammonium bicarbonate solution) was added to the samples. After the tubes were incubated at 37 °C for ~12 h, 200 μL of ACN was added, and the tubes were shaken for 10 min. The digestion solution was transferred to new microcentrifuge tubes. Then, 30 μL of 0.1% formic acid in water was added and shaken for 5 min, 200 μL of CAN was added, and the sample was again shaken for 5 min. The gel extracts were combined with the digestion solution. The samples were dried using a CentriVap concentrator (Thermo, Massachusetts, USA) and then redissolved in 0.1% formic acid in water for LC-MS/MS analysis.

The LC-MS/MS systems consisted of an EASY-nLC system (Thermo, Massachusetts, USA) and an Orbitrap Fusion Tribird mass spectrometer (Thermo, Massachusetts, USA). An Acclaim PepMap 100 column (75 μm × 15 cm, nanoViper, C18, 3 μM, 100 Å) from Thermo Fisher Scientific Inc. was used to separate the peptides. The mobile phase consisted of acetonitrile (B) and 0.1% formic acid in water that was pumped at a flow rate of 300 nL/min. The gradient started at 97% A and then linearly increased in mobile phase B from 3–8% (0–5 min), 8–18% (5–75 min), 18–28% (75–103 min), and 28–90% (103–115 min) and was then maintained at 90% (115–120 min). A data-dependent Top Speed method was used in this study. The scanning mode was orbitrap (OT)–higher energy collisional dissociation (HCD)–iontrap (IT). The instruments were operated using Tune and Xcalibur software.

MaxQuant (version: 1.6.0.13) was used to perform mass-spectrometry-based proteomics data analysis with the label-free quantification (LFQ) method. The Swiss-Prot database (human), which includes 20,412 protein sequences, was used to the identify proteins.

### Tumour xenograft model and immunohistochemistry

Female 5-week-old nude Balb/C mice were purchased from Beijing HFK Bioscience Co., Ltd. (China) and were fed in the animal house of the Institute of Hematology, Chinese Academy of Medical Sciences; the use of all animals in the experiments was approved by the Institute of Hematology, Chinese Academy of Medical Sciences. A total of 1 × 10^7^ cells/200 μL were inoculated in the axilla of nude mice. When the tumour volume reached 200 mm^3^, the mice were sacrificed, and the tumour was cut into 3 mm × 3 mm small pieces. Then, the small pieces of tumour were inoculated into the right shoulder flank of nude mice for further subculture. After the tumour was subcultured 2–3 times, the tumour was divided into several parts and inoculated into the right shoulder flank of nude mice. When the tumour volume reached ~60 mm^3^, the nude mice were randomly divided into the following four groups (5 mice/group): (1) vehicle (normal saline: NS); (2) 200 mg/kg ACT001; (3) 2.5 mg/kg cisplatin; and (4) 200 mg/kg ACT001 and 2.5 mg/kg cisplatin. ACT001 was prepared in NS and orally administered six times every 7 days for 18 days. Cisplatin was dissolved in DMSO, diluted with NS (the concentration of DMSO was 1%), and intraperitoneally injected once every 3 days for a total of six times. The body weight and tumour volume were measured every 3 days. The mice were sacrificed on the 19th day, and the tumour weights were recorded. The tumour volume (*V*) was calculated as follows: *V* = (*ab*^2^)/2, where “*a*” represents the long diameter of the tumour, and “*b*” represents the short diameter of the tumour mass. The tumours were fixed with formalin, embedded in paraffin, and cut into 5-μm sections for immunohistochemical analysis. The sections were stained with antibodies against PI3K, p-PI3K, AKT and p-AKT (antibodies were purchased from Affinity).

### Bioinformatics analysis

The differentially expressed proteins were analysed with GO and KEGG enrichment by using the ClueGO module of Cytoscape. The minimum number of genes to form an analysis result was set to three. The GO and KEGG analysis results were sorted by *P* value.

### UALCAN analysis

UALCAN database (http://ualcan.path.uab.edu) contains 31 types of cancer patients with clinical and RNA-seq data. UALCAN is an interactive portal, which can deeply analyse the relationship between the expression level of target genes in TCGA and the clinical data of patients. In this study, the expression level of PAI-1 in normal individuals and glioma patients was analysed. Meanwhile, the differences of the promoter methylation level of PAI-1 between normal individuals and glioma patients were analysed.

### CancerSEA analysis

CancerSEA (http://biocc.hrbmu.edu.cn/CancerSEA/) was used for exploring the functional status of cancer cells at the single-cell level. In gene search, single gene or gene list can be used. In this study, we analysed the relationship between PAI-1 and various cancer types and functional states. The correlation between the average expression level of PAI-1 and the functional status of glioma cells was investigated by inputting PAI-1 into the database. In addition, we analysed the distribution of PAI-1 in glioma cells by clicking on the data set name in the navigation menu.

### The Human Protein Atlas analysis

The Human Protein Atlas database aims to provide information on the distribution of all 24,000 human proteins in tissues and cells and examine the distribution and expression of each protein in 48 normal human tissues, 20 tumour tissues (including glioma), 47 cell lines and 12 blood cells using immunohistochemistry. In this study, the HPA database was used to analyse the immunohistochemical staining intensity of PAI-1 in normal brain tissue and glioma tissue and the difference of staining intensity in different glioma grades.

## Results

### PAI-1 was overexpressed in glioma

**Fig. 1:**
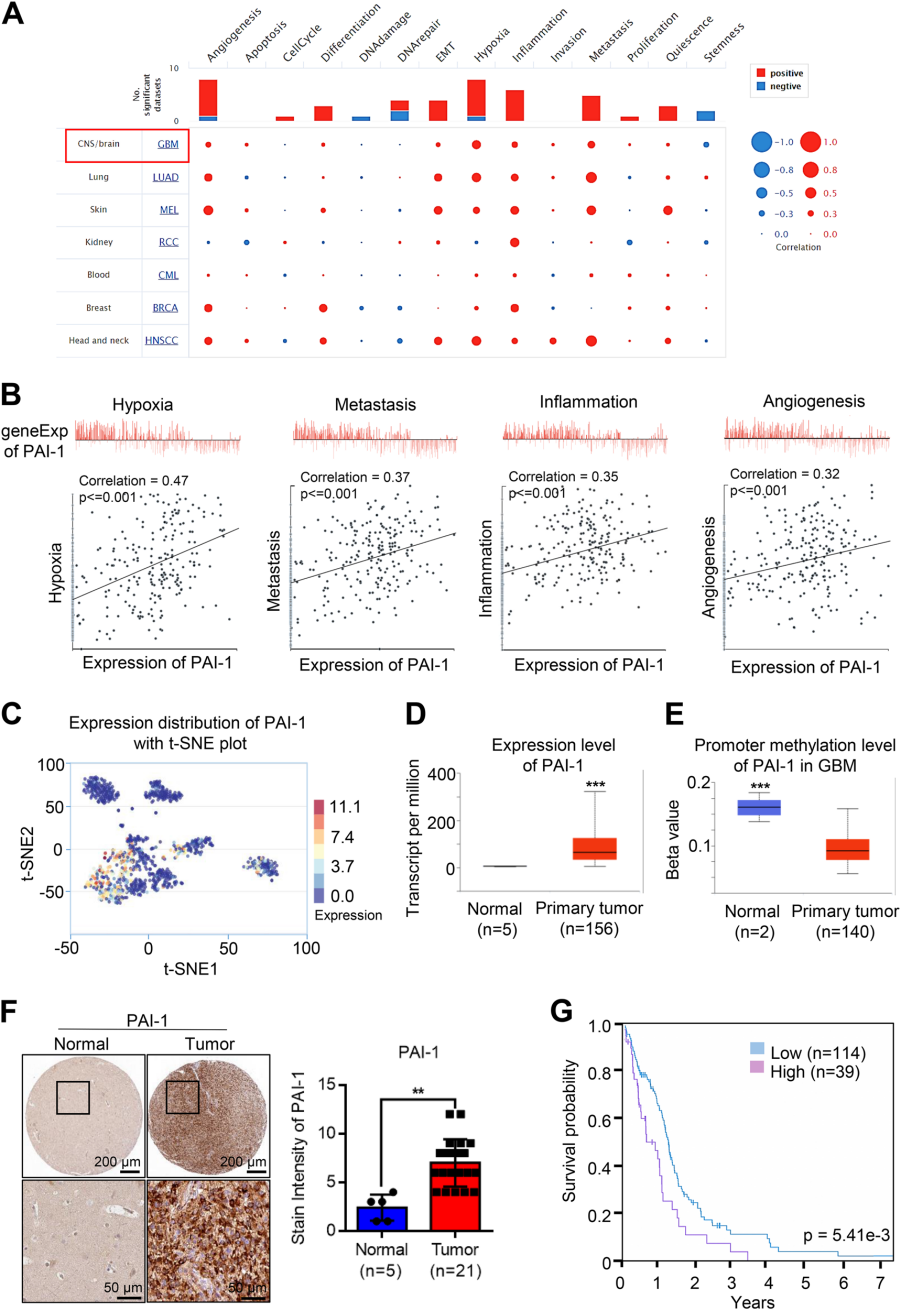
PAI-1 is upregulated in glioma cell lines and tissues. **a** Average correlations between PAI-1 expression and the functional states in different cancers. **b** Functional state analysis of PAI-1 in glioma cells. **c** Expression distribution of PAI-1 in glioma cells in the CancerSEA database. Every point represents a single cell, and the colour of the point represents the expression level of PAI-1. **d** Expression analysis of PAI-1 in normal individuals and glioma patients. Box plot shows the relative expression of PAI-1 in normal and glioma samples (UALCAN). The results revealed that PAI-1 was highly expressed in glioma patients. **e** Box plot showing relative promoter methylation level of PAI-1 in normal and glioma samples. The promoter methylation level of PAI-1 in glioma patients was reduced compared with that in healthy individuals. **f** IHC analysis of PAI-1 expression in glioma specimens and normal tissues in the human protein atlas datasets. **g** Survival analysis of PAI-1 in glioma patients. The red line represents patients with high expression, and the blue line represents patients with low expression. The *X* axis indicates overall survival time (years), and the *Y* axis indicates the survival rate. A Kaplan–Meier test was performed

The expression of PAI-1 in glioma is still unknown. To determine the expression of PAI-1 in glioma, we analysed the related functional states of PAI-1 in glioma using published gene profiling studies that are available in CancerSEA (http://biocc.hrbmu.edu.cn/CancerSEA/). From the results, PAI-1 showed the strongest correlation with glioma (Fig. 1a). Furthermore, four functional states, including hypoxia, metastasis, inflammation and angiogenesis, are significantly related to PAI-1 expression in glioma (Fig. 1b). The PAI-1 expression distribution in glioma cells with t-SNE showed that glioma cells with high PAI-1 expression tended to cluster together, which suggests that this distribution trend may promote the malignant progression of glioma (Fig. 1c). Then, we compared the mRNA levels between human normal tissues and glioma tissues using the UALCAN database (http://ualcan.path.uab.edu/index.html). The gene expression level of PAI-1 was upregulated in glioma tissues compared to normal human tissues (Fig. 1d). The promoter methylation profile of PAI-1 was downregulated in glioma tissues compared to normal tissues (Fig. 1e). We then detected the expression levels of PAI-1 protein in human normal tissues and glioma tissues with IHC (http://www.proteinatlas.org/). The results of IHC confirmed that the PAI-1 proteins were upregulated in patient glioma specimens relative to that in normal cervical tissues (Fig. 1f), which was consistent with the mRNA expression levels. In addition, we analysed the expression of PAI-1 in different glioma grades, and the analysis results showed that the PAI-1 expression gradually increased with the increase of glioma grades (Fig. S1). Moreover, the Kaplan–Meier analysis showed that high PAI-1 expression was associated with a low survival probability for glioma patients, whereas low PAI-1 expression predicted a relatively high survival probability (Fig. 1g).

### Downregulated PAI-1 affected glioma cell migration, angiogenesis and apoptosis by the PI3K-AKT signal pathway

**Fig. 2:**
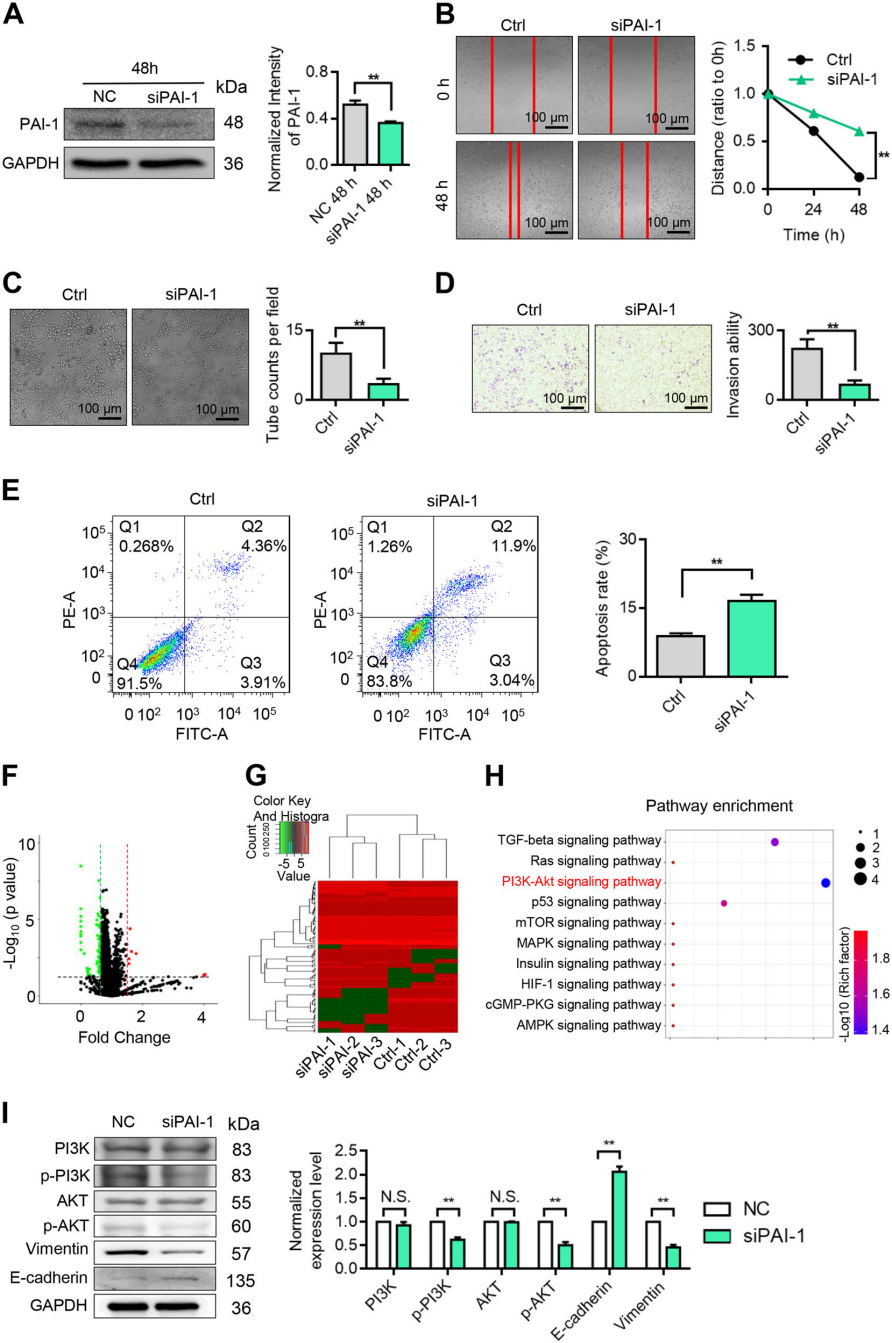
Knockdown of PAI-1 abrogates the migration and invasion ability of glioma cells. **a** Knockdown of PAI-1 in a subset of human glioma cell lines. **b** Wound healing assay results, including the control and PAI-1 knockdown groups, demonstrating the effect of PAI-1 on the migration ability of glioma cells. **c** Tube formation assay of the control and PAI-1 knockdown groups. **d** Transwell assay of the control and PAI-1 knockdown groups. **e** Apoptosis assays of the control and siPAI-1 groups were carried out by annexin V/PI staining. **f** Volcano plot of the differentially expressed proteins between the control and siPAI-1 cells. Red dots indicate upregulation, and green dots indicate downregulation of the proteins. **g** Hierarchical clustering based on the expression profiles of significantly differentially expressed proteins (DEPs) between the two groups. **h** KEGG enrichment analysis of differentially expressed proteins. **i** Western blot assay of the control and siPAI-1 cells. Data are represented as the mean ± SEM (error bars) of three independent experiments. *P < 0.05, **P < 0.01, ***P < 0.001

To verify the effect of PAI-1 on glioma cells, a PAI-1 knockdown assay was performed. After transfecting PAI-1 siRNA for 48 h, the PAI-1 protein expression was determined by western blot (Fig. 2a). To assess the effect of PAI-1 on U118MG cell migration and vascular formation ability, wound healing assays, transwell assays and VM assays were subsequently performed. The results showed that the knockdown of PAI-1 significantly inhibited the migration (Fig. 2b), vascular formation ability (Fig. 2c) and invasion (Fig. 2d) of U118 MG cells. To detect the pro-apoptotic effect of PAI-1, a cell apoptosis assay was performed by annexin V/PI staining. The results suggest that the downregulation of PAI-1 exhibited a significant pro-apoptotic effect on the U118MG cell line (Fig. 2e).

To reveal the mechanism of PAI-1 in glioma migration, angiogenesis and apoptosis, a differential proteomics assay was performed with Thermo Orbitrap Fusion after knocking down PAI-1. In total, 24 upregulated proteins and 94 downregulated proteins were chosen using *P* < 0.05 and fold change >1.5 as the cutoff criteria (Fig. 2f). The overview expression profiles of the control and siPAI-1 groups, which were exhibited after hierarchical cluster analysis, show a distinct regulating direction and a clear separation between the control and siPAI-1 groups for the DEPs (Fig. 2g). To investigate the PAI-1-related signalling pathways, pathway enrichment analysis was performed. The results indicate that the PI3K/AKT signalling pathway was the most significantly downregulated out of all ten enriched downregulated pathways. Then, the results of the pathway enrichment analysis were verified by western blot assay. The results show that the phosphorylation of PI3K and AKT was significantly reduced after the knockdown of PAI-1. The epithelial–mesenchymal transition marker protein E-cadherin was upregulated, while vimentin was downregulated after PAI-1 knockdown.

### ACT001 inhibited glioma proliferation by directly binding PAI-1

ACT001 showed excellent glioma inhibition in phase I clinical trials and was certified as an orphan drug by the FDA in the United States and in the European Union. ACT001 slowly releases MCL in vivo. The chemical structure of ACT001 is shown in Fig. S2. We summarized the reported anticancer studies of ACT001/MCL (Table S1), and the relationship between ACT001 and MCL is shown in Fig. S2. However, the molecular mechanism of ACT001 is still unknown.

**Fig. 3:**
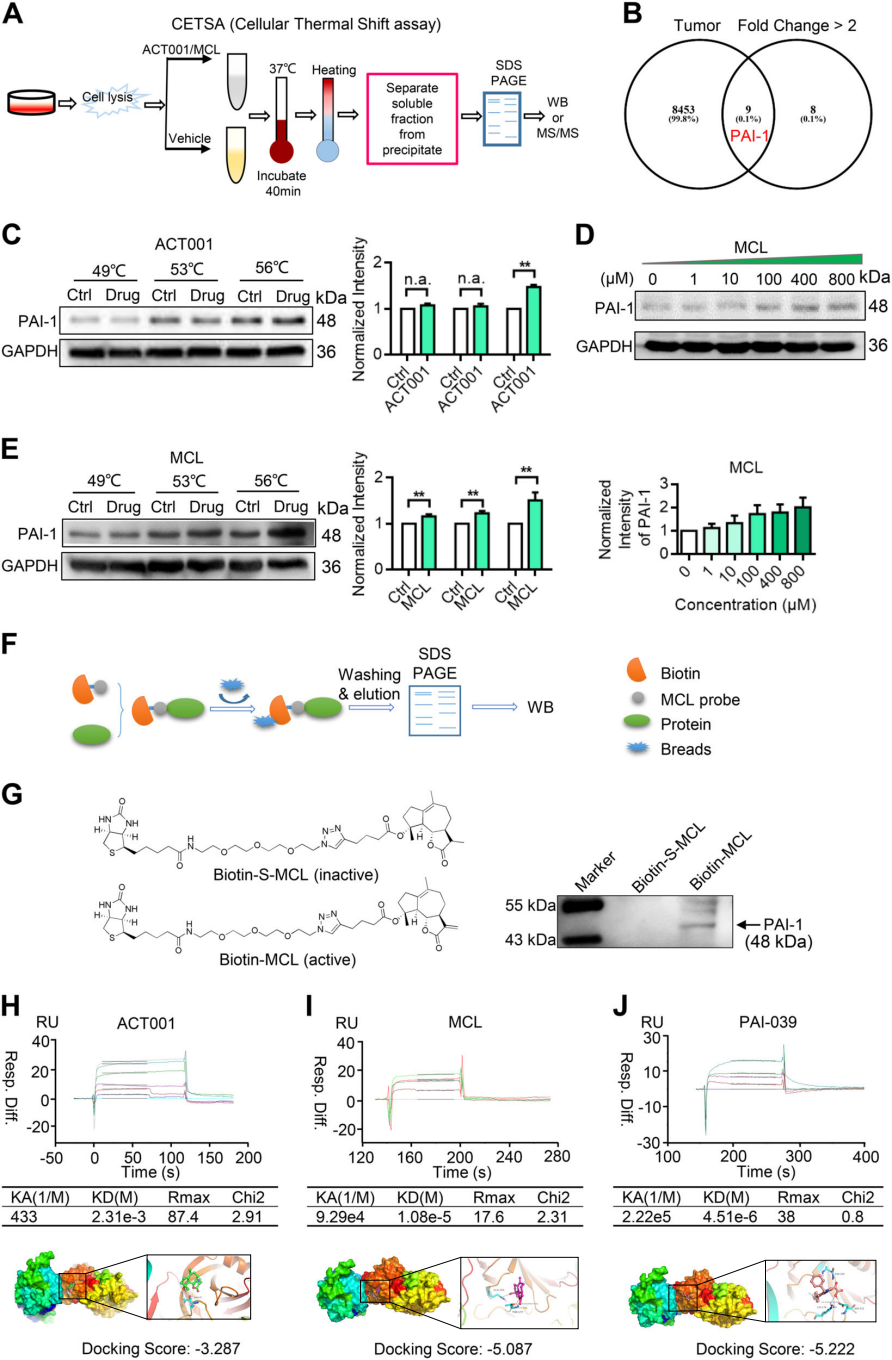
ACT001 directly targets PAI-1. **a** Flow chart of the cellular thermal shift assay. **b** Venn analysis result of the potential targets of ACT001 and tumour-associated proteins. **c** ACT001 protects the stability of PAI-1 at 56 °C. **d** MCL (the active metabolite of ACT001) stabilizes PAI-1 in a dose-dependent manner. **e** MCL stabilized PAI-1 at 49, 53 and 56 °C. **f** Diagram of the MCL probe pull-down experiment. **g** Biotin-S-MCL (inactive) and biotin-MCL (active) probe pull-down of PAI-1 from glioma cells. The results showed that only biotin-MCL can pull-down PAI-1 from glioma cells. **h** Biacore and molecular docking assays of ACT001 interacting with PAI-1. **i** Biacore and molecular docking assays of MCL interacting with PAI-1. **j** Biacore and molecular docking assay of PAI-039 interacting with PAI-1

To discover the potential targets of ACT001, TPP based on the CETSA and label-free quantitative proteomics was used (Fig. 3a). The CETSA, first described by Martinez Molina et al., is a fast method in drug target discovery that is based on the thermal stability changes after drugs bind to their target^26,27^. The authors combined the western blot technique with CETSA to directly monitor target engagement efficiently and quickly. In 2014, multiplexed quantitative MS combined with CETSA was successfully used in the discovery of drug targets^24^. The researchers named this approach TPP. LFQ is a method in MS that aims to determine the relative amount of proteins in two or more biological samples^28^. Unlike other methods (for example, multiplexed quantitative MS) for protein quantification, LFQ does not need the use of a stable isotope-containing compound to chemically bind to and label the protein. Thus, label-free quantitative proteomics is inexpensive and reliable to employ for stringent statistical validation. In our study, we combined label-free quantitative proteomics with CETSA to discover the target for ACT001.

According to the results, there were 17 proteins that changed more than twofold in the treated group compared with in the untreated group (*P* < 0.05). Nine proteins were related to tumours based on the tumour-related proteins downloaded from Swiss-Prot. PAI-1 showed the most significant change in thermal stability among these nine proteins (Fig. 3b and Table S2). Therefore, PAI-1 was speculated to be the main target of ACT001. To verify the interaction of ACT001 and PAI-1, a thermal stability assay was performed. From the results, the thermal stability of PAI-1, in terms of melting temperature, increased from 49 to 56 °C after the incubation with ACT001 (Fig. 3c). As ACT001 slowly releases MCL in vivo, the interaction of MCL and PAI-1 was analysed by the isothermal dose response (ITDR) experiment. The results indicate that the higher the MCL concentration, the better the PAI-1 thermal stability, which suggests a strong interaction between PAI-1 and MCL (Fig. 3d). The thermal stability of PAI-1 increased more significantly after the incubation with MCL, and the melting temperature increased from 49 to 56 °C (Fig. 3e). To further analyse the interaction between PAI-1 and MCL, an active probe and inactive probe of MCL was designed and synthesized (Fig. 3f, g). After pulling down the interaction protein of MCL with the active probe, a western blot assay was performed. From the results, PAI-1 was precipitated by biotin-MCL but not by biotin-S-MCL, which illustrates that PAI-1 directly interacts with MCL (Fig. 3f, g). To confirm the binding strength between PAI-1 and MCL, an SPR binding assay was conducted. The *K*_D_ values of PAI-1 with ACT001, MCL and PAI-039 (a known inhibitor of PAI-1) were 2.31 mM, 10.8 μM and 4.51 μM, respectively (Fig. 3h–j). Molecule docking calculations for PAI-1 with ACT001, MCL and PAI-039 were performed, and the docking scores were calculated by Schrodinger software. The results show that the docking scores of the three molecules were related to the *K*_D_ values. After incubation with ACT001 in 1× PBS (pH 7.4) at 37 °C overnight, the *K*_D_ value of PAI-1 with ACT001 was 9.08 μM, which was basically consistent with the *K*_D_ between MCL and PAI-1 (Fig. S3). This is because, after an overnight incubation, the active product MCL was released and interacted with PAI-1.

### ACT001 inhibits the invasion and migration ability of glioma cells by targeting PAI-1

**Fig. 4:**
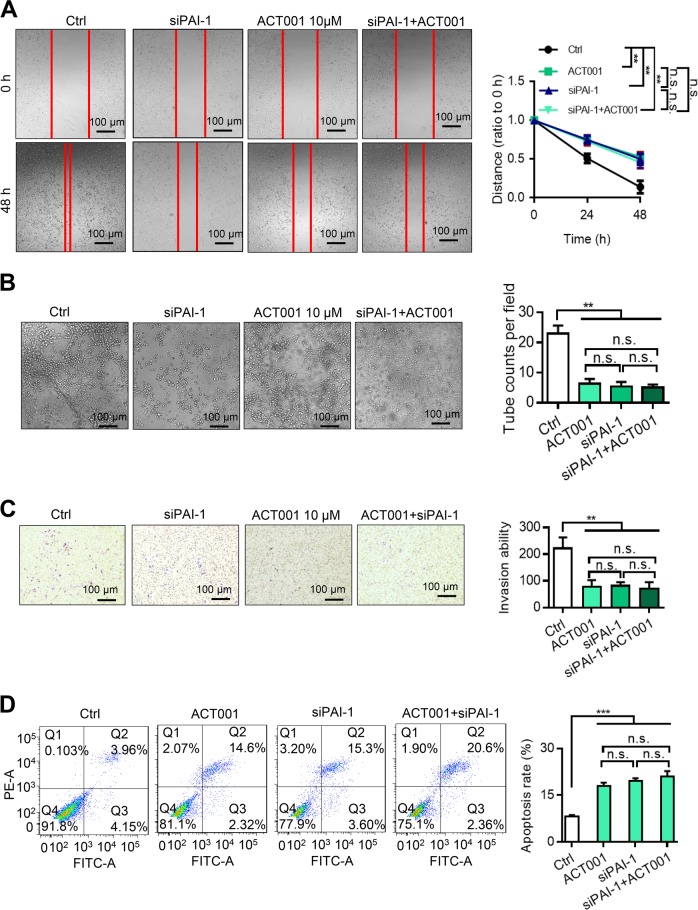
ACT001 inhibits migration, tube formation and invasion and induces apoptosis in glioma cells. **a**–**c** ACT001 treatment can significantly inhibit the migration, tube formation and invasion capabilities of glioma cells. A transfection of siRNA PAI-1 did not significantly alter the inhibitory effect of ACT001. **d** Effect of ACT001 on glioma cell apoptosis. The results were obtained from three independent experiments, and each experiment was performed in triplicate. Data are represented as the mean ± standard error of the mean (*P < 0.05, **P < 0.01)

To verify whether ACT001 inhibited glioma by targeting the PAI-1 protein, wound healing assays, VM assays and transwell assays were performed. The migration, invasion and vascular formation ability of U118MG cells were inhibited after treatment with 10 μM ACT001. When PAI-1 was knocked down and treated with 10 μM ACT001, there were no significant changes in cell migration, invasion and vascular formation ability compared with the knockdown group and the ACT001 treatment group (Fig. 4a–c). These results suggest that the knockdown of PAI-1 inhibited the effect of ACT001. To investigate the role of PAI-1 in U118MG cell apoptosis, the cell apoptosis percentage was detected by flow cytometry. As shown in Fig. 4d, the total apoptosis rate of the ACT001 (10 μM) group was 17.88 ± 1.13%, the total apoptosis rate of the siRNA PAI-1 group was 19.62 ± 0.79%, and the total apoptosis rate of the ACT001 (10 μM)/siRNA PAI-1 group was 21.05 ± 1.65%. There were significant differences (*P* < 0.001) compared with the control group (8.15 ± 0.47%). These results indicate that ACT001 can induce apoptosis in U118MG cells and that the inhibition effects of ACT001 cannot be enhanced by PAI-1 knockdown. This may be because the target protein of ACT001 was knocked down, so the effect of ACT001 was weakened.

### ACT001 inhibits glioma cells by downregulating the PI3K/AKT pathway

**Fig. 5:**
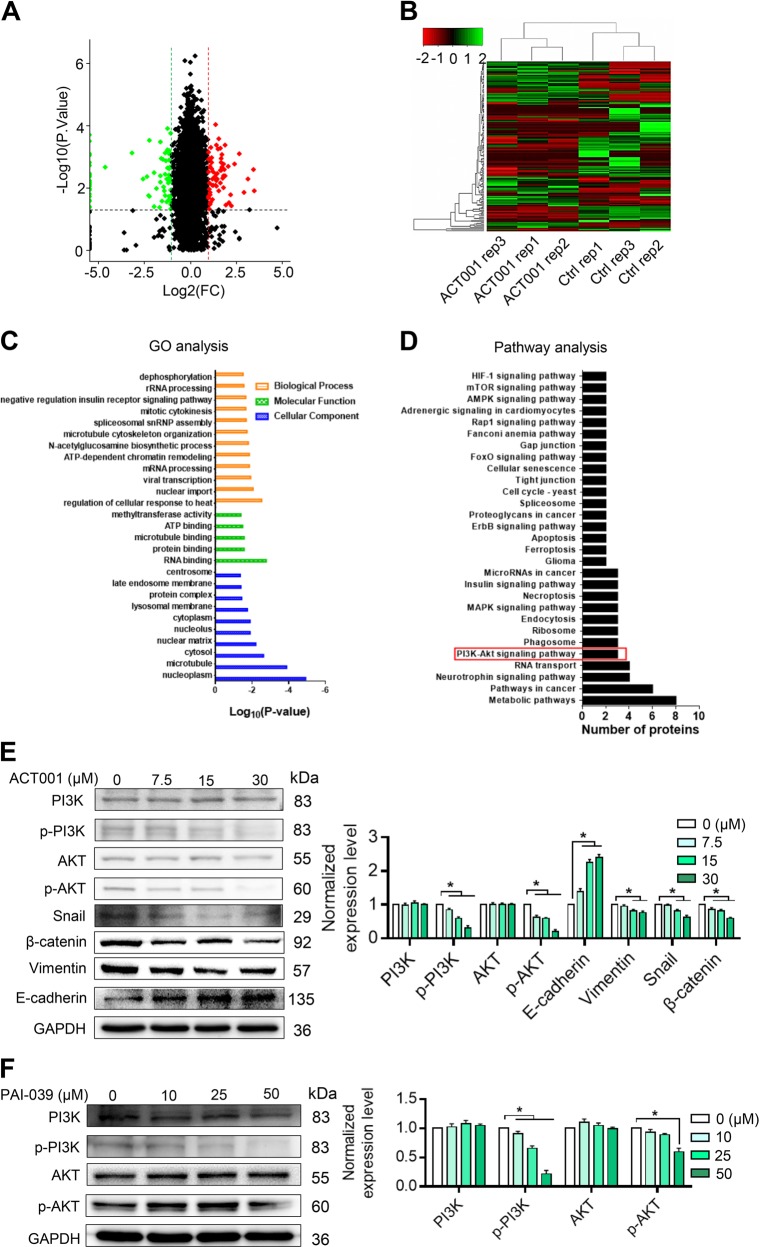
ACT001 inhibits glioma cells through the PI3K/AKT signalling pathway. **a** Volcano plot of the differentially expressed proteins between the control and 20 μM ACT001-treated groups. Red dots indicate upregulation, and green dots indicate downregulation of the proteins. **b** Hierarchical clustering based on the expression profiles of significantly differentially expressed proteins in the control and 20 μM ACT001-treated groups. **c** GO analysis of the differentially expressed proteins. **d** KEGG analysis of the differentially expressed proteins. **e** The PI3K/AKT pathway, β-catenin, Snail, E-cadherin and vimentin were detected in glioma cells before (Con) and 24 h after ACT001 treatment at the indicated concentrations. **f** The PI3K/AKT pathway was detected in glioma cells before (Con) and 24 h after PAI-039 treatment at the indicated concentrations. The results were obtained from three independent experiments, and each experiment was performed in triplicate. Data are represented as the mean ± standard error of the mean (*P < 0.05, **P < 0.01)

To determine the molecular pathway of ACT001, a proteomics assay was subsequently performed in U118MG cells. A fold change >1.5 or <0.06 and a *P* < 0.05 were used as the cutoff criteria (Fig. 5a). The clustering analysis results showed that ACT001 significantly changed the protein expression of U118MG cells (Fig. 5b & Table S3). GO enrichment analysis, including analysis of biological processes, molecular functions and cellular components, was performed. The results show that mitotic cytokinesis, microtube cytoskeleton organization, ATP-dependent chromatin remodelling and nuclear import were inhibited by ACT001 (Fig. 5c). The results of the pathway enrichment analysis show that ACT001 significantly inhibited the PI3K/AKT signalling pathway (Fig. 5d). The PI3K/AKT pathway plays a key role in glioma cell invasion. After treatment with ACT001 for 48 h, the p-PI3K and p-AKT protein levels in glioma cells were significantly reduced compared to the PAI-039 treatment group (a PAI-1 inhibitor) (Fig. 5e, f). The results above show that ACT001 acts as a PAI-1 inhibitor by inhibiting the phosphorylation of PI3K and AKT.

### ACT001 enhances the antitumour effect of cisplatin in vitro and in vivo

**Fig. 6:**
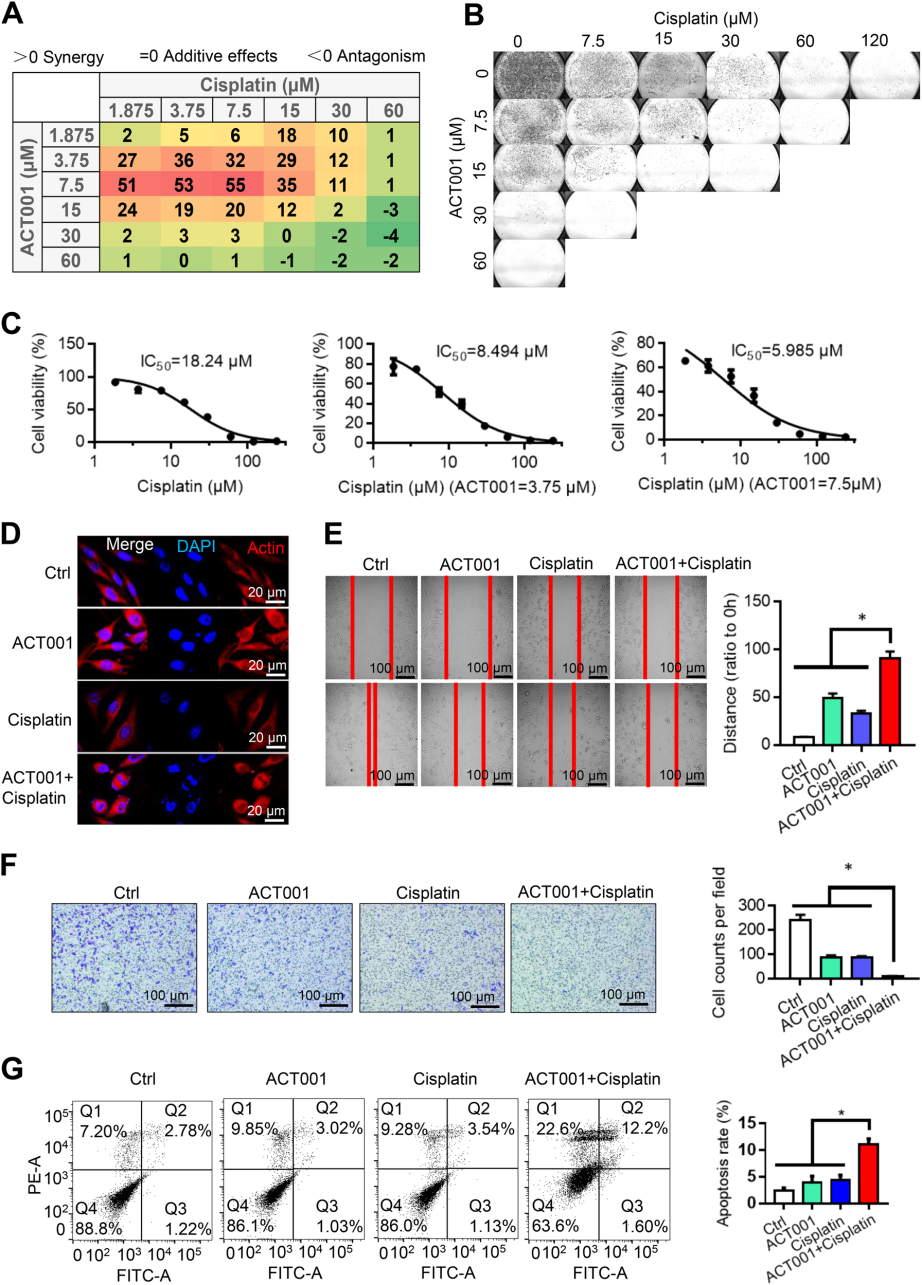
ACT001 enhances the effect of cisplatin in vitro. **a** MTT assay of cells treated with ACT001 or cisplatin or both. **b** Cell survival assay with crystal violet staining of cells treated with ACT001 or cisplatin or both. **c** A synergistic effect was observed with a combination of ACT001 and cisplatin. **d** The cytoskeleton of glioma was stained in the ACT001, cisplatin and combination ACT001 and cisplatin groups. **e**–**g** Wound healing, transwell and apoptosis assays of the ACT001, cisplatin and combination ACT001 and cisplatin groups

Cisplatin resistance was associated with PAI-1 expression, and PI3K/AKT pathway inhibitors act synergistically with cisplatin-based chemotherapy or radiotherapy. Therefore, we suspected that ACT001 combined with cisplatin might demonstrate a good antitumour effect in U118MG cells. The combination of ACT001 and cisplatin was determined by the MTT assay and crystal violet staining assay. The synergistic effect was assessed using the Bliss independence model. According to the results, ACT001 and cisplatin showed synergistic efficacy at a wide range of concentrations, and the maximum synergistic effect was 7.5 μM ACT001 and 7.5 μM cisplatin (Fig. 6a, b). The 50% inhibitory concentration (IC_50_) of cisplatin was 18.24, 8.494 and 5.985 μM when combined with ACT001 at concentrations of 0, 3.75 and 7.5 μM, respectively (Fig. 6c). As a result, we selected 7.5 μM ACT001 and 7.5 μM cisplatin for the following combination experiments. The results showed that cisplatin and ACT001 synergistically induce changes in cell morphology and cell nucleus integrity (Fig. 6d). The results of the wound healing assay indicate that the combination of ACT001 and cisplatin reduced cell migration compared to either drug alone (Fig. 6e). The results of the transwell assay suggested that the combination of ACT001 and cisplatin significantly reduced cell invasion compared with the ACT001 treatment group or cisplatin treatment group (Fig. 6f). Flow cytometry analysis was performed to determine the combined effect of ACT001 and cisplatin on cell apoptosis. The results showed that the apoptosis rate was 13.7 ± 1.08% after ACT001/cisplatin treatment, 4.5 ± 0.62% after ACT001 treatment, 5.1 ± 0.99% after cisplatin treatment and 4.19 ± 0.50% after vehicle treatment. The apoptosis rate of the ACT001/cisplatin group was significantly increased compared with that of the vehicle, ACT001 or cisplatin treatment groups (*P* < 0.01) (Fig. 6g). These results suggest that ACT001 combined with cisplatin increased the apoptosis of U118MG cells over either drug alone.

**Fig. 7:**
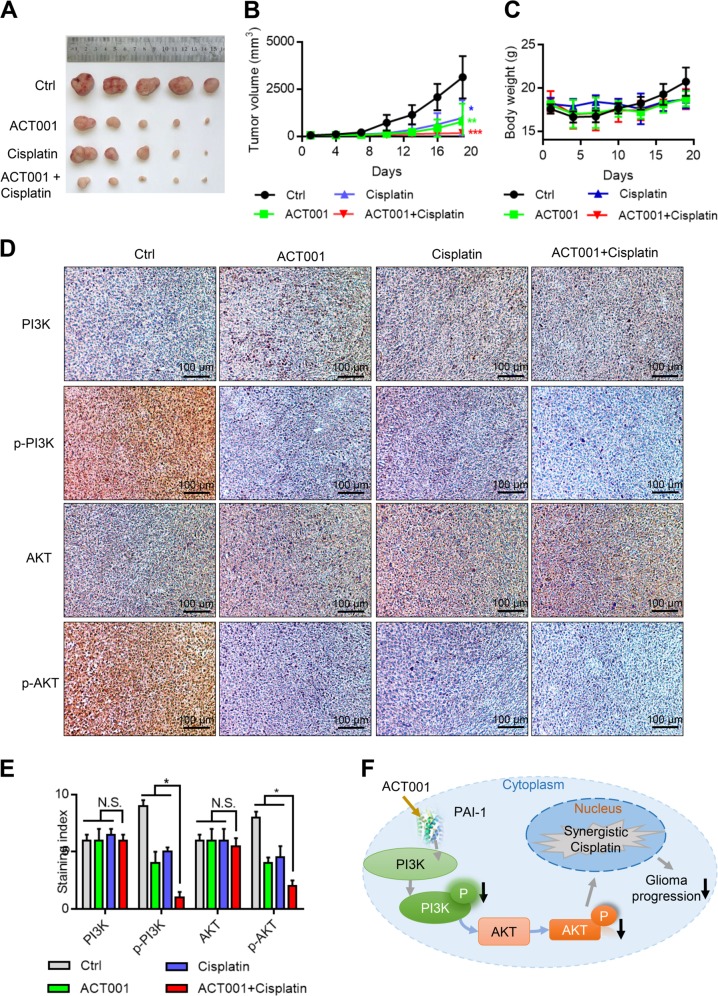
ACT001 enhances the antitumour effect of cisplatin in vivo. **a** Xenograft assays in the mice treated with control, ACT001, cisplatin and ACT001 + cisplatin. **b** Tumour volume changes in the mice treated with control, ACT001, cisplatin and ACT001 + cisplatin. **c** Body weight changes in the mice treated with control, ACT001, cisplatin and ACT001 + cisplatin. **d** IHC analysis of the PI3K, p-PI3K, AKT and p-AKT levels. Compared with the control group, the staining of p-PI3K and p-AKT was lighter in the ACT001 treatment groups. In the ACT001 + cisplatin treatment group, the staining of p-PI3K and p-AKT was lighter than in the ACT001-treated group. **e** Immunohistochemical staining of PI3K, p-PI3K, AKT and p-AKT. **f** Mechanism of how ACT001 inhibits tumour progression. The results show that ACT001 inhibits tumour progression through the PAI-1/PI3K/AKT pathway

To further investigate the combined effect of ACT001 and cisplatin in vivo, we used an U118 xenograft model. The mice were treated with cisplatin (2.5 mg/kg, once every 3 days, intraperitoneal injection), ACT001 (200 mg/kg, once daily, oral administration) or the combination of ACT001 and cisplatin. The xenografts in the combination treatment group exhibited a relatively slower growth rate than those in the other groups. The tumour size and tumour weight in the combination treatment group were remarkably reduced compared with those in the other groups (Fig. 7a, b, Table S4). In addition, there was no significant difference in body weight between the groups (Fig. 7c). According to the Bliss independence model, the combination index was 2.67, which was >0, indicating that the drug combination is better than each drug alone. Furthermore, the PI3K, p-PI3K, AKT and p-AKT protein levels in the tumour xenografts were analysed by IHC assay. The results showed that the expression of PI3K and AKT was not changed, but the expression of p-PI3K and p-AKT was reduced in the ACT001, cisplatin and combination groups (Fig. 7d). In addition, the tumour xenografts in the combination treatment group showed robust p-PI3K and p-AKT expression compared with that of the other groups (Fig. 7e).

In conclusion, PAI-1 promoted glioma cell proliferation through the PI3K/AKT signalling pathway. ACT001 exerts its anti-glioma effect by directly binding PAI-1 (Fig. 7f).

## Discussion

PAI-1, a protease inhibitor, is significantly associated with poor clinical outcomes in glioma. However, the molecular mechanism of PAI-1 on glioma cells is still unclear, and there are no drugs that target PAI-1. Given the critical role of PAI-1 in tumour proliferation, invasion and drug resistance, we studied the function of PAI-1 in glioma in the current study. We confirmed the overexpression of PAI-1 in glioma patients and investigated the pharmacological mechanism of ACT001 by directly targeting PAI-1.

The PI3K/AKT pathway is critical for controlling most hallmarks of cancer. Numerous studies have indicated that tumourigenesis is partly caused by alterations in the PI3K/AKT pathway^29–33^. AKT, an important signalling molecule downstream of PI3K, plays a key role in the invasion of cancer cells by regulating MMP-2, which regulates the migration and metastasis of cancer cells by degrading the extracellular matrix^34,35^. Our experimental results showed that PAI-1 can regulate the PI3K/AKT signalling pathway. Therefore, the inhibition of PAI-1 could inhibit tumour cell proliferation, invasion and migration through the downregulation of the PI3K/AKT pathway.

The biggest challenge and difficulty in glioma treatment lies in the ability of the drug to penetrate the blood–brain barrier to enter the brain. ACT001, which can penetrate the blood–brain barrier and accumulate in the brain, was first discovered and developed by our group^10^. The concentration of ACT001 in the brain is 1.8 times higher than that in the blood. The concentration of the best drug for the treatment of glioma, temozolomide, was only 0.4 times higher in the brain than that in the blood. However, the mechanism of ACT001 is still unclear, which restricts the clinical application of ACT001 and limits the selection of patients for clinical experiments. In this study, we demonstrated that ACT001 exhibited an antitumour effect by directly targeting PAI-1 and further inhibiting the PI3K/AKT pathway in the U118 cell line in a dose-dependent manner. Our results also indicated that ACT001 inhibited glioma cell proliferation, migration, angiogenesis and apoptosis in the U118 cell line. These results provided evidence that ACT001 is an effective anticancer agent in glioma therapy as a novel PAI-1 inhibitor.

The systemic treatment of glioma aims to increase tumour cell cytotoxicity, reduce normal cell damage, palliate symptoms and prolong survival. Cisplatin has been used for glioma treatment^36^. However, due to the dose-dependent toxicity and drug resistance of platinum-based compounds in the clinic, the efficacy of cisplatin in glioma treatment is very limited^9^. To obtain a sustainably significant effect, it is necessary to increase the dose of the drugs. However, an increased drug dose often led to increased toxicity. Therefore, the development of new therapeutic strategies to overcome the toxicity and drug resistance of platinum-based compounds is the top priority in the treatment of glioma. It was not enough to stabilize the initial subcutaneous tumour response with ACT001 or cisplatin administration alone. In this study, the in vivo animal results showed that the combination of ACT001 and cisplatin significantly decreased U118 tumour volumes and tumour weight compared to the each drug treatment alone, which suggested that ACT001 combined with platinum-based compounds has potential therapeutic value in targeting glioma.

Effective therapeutic targets and drugs for glioma are urgently needed in the clinic. We provided compelling preclinical evidence that PAI-1 inhibitors are effective as monotherapy agents and in combination with cisplatin in glioma models. Considering that drug resistance eventually develops in almost all glioma patients, the discovery of a PAI-1 inhibitor has important clinical significance. However, this study still has some shortcomings. First, brain glioma cell line types should be added in subsequent studies to verify the importance of PAI-1 in many cell lines. Second, in addition to PAI-1, there may be other therapeutic targets for brain glioma of ACT001, which need to be further explored. Finally, ACT001 combined with cisplatin in animal models would be more convincing if the in situ glioma model could be introduced. In summary, ACT001 is currently undergoing clinical trials in glioma treatment. We demonstrated for the first time that targeting PAI-1 may contribute to the treatment of glioma. PAI-1 may be a candidate biomarker for glioma, and patients with high PAI-1 expression may benefit from PAI-1 inhibitors. These results supported the studies on PAI-1 as a biomarker of glioma and further support the clinical development of ACT001 as a drug for glioma treatment for patients in phase II clinical trials.

## Supplementary information

Supplementary data
