# Supplementary material for: ACT001, a novel PAI-1 inhibitor, exerts synergistic effects in combination with cisplatin by inhibiting PI3K/AKT pathway in glioma
Source: Cell Death Dis. 2019 Oct 7;10(10):757. doi: 10.1038/s41419-019-1986-2 (PMC6779874; doi:10.1038/s41419-019-1986-2)
Supplement: Supplementary file 1 — Supplementary data [file 41419_2019_1986_MOESM1_ESM.docx]

**ACT001, a novel PAI-1 inhibitor, exerts synergistic effects in combination with cisplatin by inhibiting PI3K/AKT pathway in glioma**

**Running title：ACT001 targets PAI-1 to inhibit glioma**

Xiaonan Xi^1,2^, Ning Liu^2^, Qianqian Wang^2^, Yahui Chu^1,2^, Zheng Yin^1^, Yahui Ding^1,2*^, Yaxin Lu^1,2*^

^1^ College of Pharmacy, Nankai University, Tianjin, 300350, P. R. China

^2^ State Key Laboratory of Medicinal Chemical Biology, Nankai University, Tianjin, 300350, P. R. China

### ^*^Corresponding authors: Yaxin Lu and Yahui Ding

### Yaxin Lu: E-mail: [yaxinlu@nankai.edu.cn](mailto:yaxinlu@nankai.edu.cn), Fax: +86-22-23507760; Tel: +86-22-23508371, College of Pharmacy, The State Key Laboratory of Medicinal Chemical Biology, Nankai University, Tianjin 300350, P. R. China.

### Yahui Ding: E-mail: [017095@nankai.edu.cn](mailto:017095@nankai.edu.cn), Fax: +86-22-23507760; Tel: +86-22-23508371, College of Pharmacy, The State Key Laboratory of Medicinal Chemical Biology, Nankai University, Tianjin 300350, P. R. China.

### None of these details should not be published.

**Keywords:** ACT001; glioma; PAI-1/PI3K/AKT; cisplatin

**Supplementary tables**

**Table S1.** Anticancer studies reporting on ACT001/MCL.

| **Cancers** | **Mechanism** | **Number of references** |
| --- | --- | --- |
| Leukemia | NF-κB pathway/ROS | 2 |
| Breast cancer | KLF4-mediated resistance | 1 |
| Breast cancer | Dynamin-related protein 1(ROS–mitochondrial apoptotic pathway) | 1 |
| Glioma | Apoptosis (Bcl-2) | 1 |
| Colorectal cancer | NF-κB pathway | 1 |

**Table S2.** Nine proteins showed significant changes in the thermal stability experiments.

| **Protein IDs** | **Protein names** | **Gene names** | **T(℃)** | **Fold change** |
| --- | --- | --- | --- | --- |
| P05121 | Plasminogen activator inhibitor 1 | PAI-1 (SERPINE1) | 53 | 2.8 |
| P08397 | Porphobilinogen deaminase | HMBS | 53 | 2.2 |
| Q15651 | High mobility group nucleosome-binding domain-containing protein 3 | HMGN3 | 53 | 2.2 |
| Q9UQ35 | Serine/arginine repetitive matrix protein 2 | SRRM2 | 53 | 2.1 |
| Q15651 | High mobility group nucleosome-binding domain-containing protein 3 | HMGN3 | 49 | 2.0 |
| Q7L2J0 | 7SK snRNA methylphosphate capping enzyme | MEPCE | 49 | 2.8 |
| Q12931 | Heat shock protein 75 kDa, mitochondrial | TRAP1 | 45 | 2.3 |
| Q9UHB9 | Signal recognition particle subunit SRP68 | SRP68 | 45 | 2.1 |
| Q7Z3B4 | Nucleoporin p54 | NUP54 | 41 | 2.2 |
| Q9BYB4 | Guanine nucleotide-binding protein subunit beta-like protein 1 | GNB1L | 41 | 2.1 |

**Table S3. Differentially expressed proteins in ACT001 treated glioma cells.**

| Protein IDs | LFQ intensity Ctrl-1 | LFQ intensity Ctrl-2 | LFQ intensity Ctrl-3 | LFQ intensity Drug-1 | LFQ intensity Drug-2 | LFQ intensity Drug-3 |
| --- | --- | --- | --- | --- | --- | --- |
| Q6P9F0 | 0 | 0 | 393870 | 4535000 | 0 | 0 |
| Q9UGY1 | 4692900 | 0 | 0 | 25675000 | 12143000 | 13711000 |
| Q70HW3 | 974760 | 0 | 0 | 2302100 | 4190700 | 2283100 |
| Q6Y1H2 | 867620 | 0 | 0 | 1448400 | 1770100 | 3450200 |
| Q01628 | 0 | 0 | 2189900 | 3195300 | 8714900 | 2193900 |
| Q9H3K2 | 675510 | 0 | 0 | 964310 | 1371000 | 1339400 |
| Q93077 | 1774400 | 0 | 0 | 2045400 | 4490600 | 2042000 |
| O00391 | 418080 | 0 | 0 | 782590 | 377070 | 806260 |
| P04156 | 0 | 2407700 | 0 | 3305500 | 3854200 | 3244400 |
| Q8NHV4 | 370860 | 0 | 0 | 524160 | 363480 | 695550 |
| Q9NVR2 | 317270 | 0 | 0 | 406270 | 442240 | 369710 |
| Q9BXK1 | 0 | 777650 | 0 | 822740 | 1363300 | 747710 |
| Q8N9T8 | 491890 | 0 | 0 | 639290 | 537340 | 667880 |
| Q96FZ2 | 936890 | 0 | 0 | 1172200 | 880330 | 1394700 |
| Q66K79 | 0 | 0 | 491470 | 590990 | 415040 | 749890 |
| Q6P6C2 | 786210 | 0 | 0 | 1201000 | 817980 | 786350 |
| P16083 | 0 | 2379300 | 0 | 2531300 | 2467500 | 3331900 |
| Q8NG68 | 881720 | 0 | 0 | 832470 | 1107200 | 1123800 |
| P61024 | 1497500 | 0 | 0 | 0 | 1424000 | 3614200 |
| Q29960 | 0 | 0 | 3283500 | 3641800 | 3081500 | 4214500 |
| P50750 | 0 | 0 | 1378100 | 1964300 | 1359600 | 1190900 |
| Q9H2P0 | 0 | 0 | 907300 | 991400 | 875620 | 1084900 |
| Q9C005 | 0 | 2231100 | 0 | 2228300 | 2068000 | 2859400 |
| Q96I25 | 611650 | 0 | 0 | 1066400 | 0 | 887900 |
| O75582 | 478970 | 0 | 0 | 496370 | 497360 | 505280 |
| Q9Y2C4 | 0 | 0 | 1007900 | 885880 | 907260 | 1352000 |
| Q9BPZ7 | 0 | 0 | 1328600 | 1265700 | 1247700 | 1411800 |
| Q8IWF2 | 0 | 919560 | 0 | 1054700 | 730370 | 926010 |
| P58557 | 1061200 | 0 | 920200 | 1470600 | 1577600 | 2617500 |
| O95758 | 1343900 | 0 | 0 | 1601900 | 1129400 | 1012400 |
| Q9BXS6 | 0 | 748450 | 0 | 925520 | 559620 | 587760 |
| P02675 | 658140 | 1159700 | 0 | 1750700 | 1974900 | 1278600 |
| O75691 | 382360 | 491660 | 472060 | 1175600 | 691470 | 1837700 |
| P22090 | 0 | 0 | 2612200 | 1819500 | 2118600 | 3116400 |
| Q14980 | 0 | 732090 | 1347700 | 1333700 | 2365900 | 1881300 |
| P62857 | 5790700 | 6204100 | 4723000 | 15197000 | 14374000 | 15137000 |
| P62273 | 4192000 | 3726400 | 3328800 | 9433400 | 10628000 | 9731000 |
| Q9UBT7 | 0 | 1834700 | 0 | 1594300 | 1462800 | 1780900 |
| Q9GZT9 | 1311600 | 0 | 0 | 978060 | 1210900 | 1161800 |
| O15523 | 2449800 | 2029600 | 1562800 | 4201200 | 3777100 | 6757200 |
| P27448 | 0 | 0 | 654300 | 871470 | 705440 | 0 |
| Q96PK6 | 2452400 | 2130500 | 1788600 | 5777000 | 4141800 | 5379700 |
| Q86YR5 | 3820500 | 11639000 | 0 | 17548000 | 12402000 | 6331900 |
| Q9Y2I1 | 771600 | 0 | 691440 | 1324700 | 988370 | 1077800 |
| Q8N8R5 | 1373600 | 0 | 0 | 2094800 | 0 | 1086800 |
| Q9BV38 | 795600 | 0 | 0 | 606760 | 495980 | 708460 |
| O94776 | 4879800 | 5471900 | 4373000 | 12190000 | 10203000 | 10814000 |
| Q9UQ90 | 2374500 | 3796600 | 0 | 0 | 6919400 | 6875400 |
| Q9BV10 | 638930 | 914990 | 0 | 1251500 | 986330 | 1205500 |
| Q86U44 | 666430 | 492680 | 626510 | 1302900 | 1329200 | 1274300 |
| Q9UBP0 | 0 | 2627900 | 2654400 | 3510000 | 4308400 | 3613900 |
| Q16637 | 0 | 2987800 | 0 | 3373800 | 2973300 | 0 |
| Q92922 | 843590 | 0 | 0 | 767310 | 1022600 | 0 |
| Q9UGM6 | 4907700 | 2746100 | 0 | 5133600 | 5429700 | 5636400 |
| P61962 | 2470100 | 2121600 | 4113700 | 5198400 | 6735300 | 6424100 |
| Q9H1A3 | 0 | 1843600 | 2303700 | 4481800 | 1349500 | 2882200 |
| Q13191 | 0 | 800640 | 0 | 694520 | 962480 | 0 |
| P62306 | 3471600 | 2648000 | 3038000 | 6569600 | 5872000 | 6413800 |
| Q9Y2U8 | 1496400 | 1857000 | 1311100 | 3095400 | 2217300 | 4266800 |
| O14763 | 1711000 | 792360 | 0 | 1636500 | 2177100 | 1284700 |
| Q96S82 | 4711400 | 1835500 | 3693300 | 7216700 | 6285000 | 7309900 |
| Q9P2N5 | 1038900 | 0 | 793760 | 1970300 | 1743000 | 0 |
| Q9Y223 | 2886400 | 3043600 | 2377200 | 5984500 | 4402900 | 6348500 |
| Q8N1F8 | 0 | 0 | 852070 | 648480 | 1066500 | 0 |
| Q9H4L7 | 1395200 | 1227900 | 1145200 | 803710 | 1068700 | 0 |
| Q9BXW9 | 0 | 3195600 | 1194800 | 0 | 0 | 2141000 |
| Q9P253 | 14366000 | 14054000 | 14370000 | 6210200 | 7531600 | 7124100 |
| Q9UPW5 | 923010 | 694490 | 0 | 0 | 787090 | 0 |
| Q16878 | 43983000 | 29882000 | 24174000 | 0 | 10961000 | 36153000 |
| P13473 | 41392000 | 51853000 | 46249000 | 21520000 | 20831000 | 24244000 |
| Q9UJS0 | 7512900 | 8974700 | 10140000 | 3631300 | 4033800 | 5042300 |
| Q14678 | 1608700 | 1856700 | 1531400 | 1087700 | 1296600 | 0 |
| Q8NBT2 | 8741800 | 35184000 | 22321000 | 11176000 | 7797900 | 12611000 |
| O95159 | 7825600 | 9167200 | 9225800 | 4786100 | 4006500 | 3607100 |
| Q96BZ9 | 406220 | 582780 | 0 | 464930 | 0 | 0 |
| Q16777 | 5571800 | 2381400 | 2953400 | 2093000 | 2948400 | 0 |
| P28290 | 2916200 | 2231000 | 2519900 | 1337100 | 1102500 | 1066000 |
| O14936 | 384630 | 455900 | 585720 | 417170 | 232850 | 0 |
| O43815 | 13653000 | 14086000 | 15378000 | 5609000 | 6193000 | 7807700 |
| Q9NVT9 | 4996000 | 3197400 | 8687000 | 2045800 | 2429600 | 3166200 |
| P09619 | 7308000 | 7551600 | 6420500 | 4518200 | 2399500 | 2694800 |
| Q9Y6D6 | 2537900 | 1420400 | 2675700 | 1776800 | 1208400 | 0 |
| Q92562 | 0 | 1879300 | 2588200 | 877510 | 0 | 1117100 |
| O75648 | 0 | 1079300 | 0 | 0 | 0 | 472050 |
| Q7Z3J2 | 15175000 | 14073000 | 16587000 | 5352000 | 6772800 | 7554300 |
| Q9H6E5 | 0 | 801560 | 1042500 | 790330 | 0 | 0 |
| Q9H492 | 2292300 | 8143800 | 7116000 | 2627600 | 2241500 | 2580200 |
| Q08AD1 | 2955000 | 2791500 | 3503100 | 2329000 | 1595400 | 0 |
| Q9UM22 | 12235000 | 13576000 | 17441000 | 3813800 | 4782200 | 9340900 |
| Q96AQ6 | 11124000 | 12334000 | 12599000 | 4361100 | 4840800 | 5631900 |
| Q10472 | 1161800 | 1395000 | 1002200 | 629380 | 0 | 806570 |
| O14530 | 10135000 | 10250000 | 10549000 | 4499800 | 4236400 | 3510000 |
| P35568 | 633180 | 662660 | 1030400 | 0 | 914360 | 0 |
| Q9BSU1 | 1637600 | 0 | 1319100 | 0 | 0 | 1153900 |
| Q12982 | 2980100 | 4381000 | 4021500 | 1235000 | 2093400 | 1029900 |
| P49137 | 2300200 | 1682700 | 1606800 | 1188300 | 837570 | 0 |
| P55011 | 1014500 | 1106600 | 1273500 | 0 | 0 | 1215300 |
| Q5T011 | 0 | 2894400 | 2595900 | 0 | 847530 | 1066800 |
| Q9UGJ1 | 698280 | 1604900 | 4166500 | 1092400 | 0 | 1054000 |
| Q9P2X3 | 0 | 1301600 | 1802800 | 0 | 1024400 | 0 |
| P52948 | 1103000 | 528230 | 1182500 | 0 | 0 | 920370 |
| Q8N1I0 | 1637200 | 1239900 | 1264500 | 1336200 | 0 | 0 |
| Q96RD7 | 3404500 | 2007200 | 1679400 | 0 | 2263100 | 0 |
| Q96SL1 | 2142300 | 1949500 | 2600800 | 0 | 1895200 | 0 |
| Q9Y4F4 | 11377000 | 17987000 | 19831000 | 0 | 12683000 | 0 |
| Q9H4I2 | 1254900 | 1062800 | 1208400 | 902270 | 0 | 0 |
| Q9P2Y5 | 2303600 | 2495300 | 1668700 | 1397000 | 0 | 0 |
| Q8TBE9 | 1776200 | 0 | 2063900 | 0 | 819850 | 0 |
| Q99574 | 1278300 | 277450 | 1605900 | 0 | 0 | 650150 |
| Q9Y426 | 0 | 1397500 | 1498300 | 0 | 0 | 577230 |
| Q9Y4F3 | 3940200 | 4427400 | 4217700 | 2152200 | 0 | 0 |
| Q9H900 | 2888100 | 3516700 | 4373600 | 1210900 | 0 | 0 |
| Q5HYC2 | 0 | 4737700 | 0 | 192920 | 0 | 0 |
| A6NHL2 | 0 | 0 | 3058100 | 0 | 0 | 0 |
| O14569 | 0 | 488550 | 0 | 0 | 0 | 0 |
| O43709 | 0 | 2742300 | 2546100 | 0 | 0 | 0 |
| O95149 | 1540800 | 0 | 1403500 | 0 | 0 | 0 |
| P13051 | 0 | 1285200 | 0 | 0 | 0 | 0 |
| P16383 | 0 | 2376600 | 0 | 0 | 0 | 0 |
| P18583 | 0 | 0 | 1956700 | 0 | 0 | 0 |
| P49247 | 2751900 | 0 | 2936200 | 0 | 0 | 0 |
| P54278 | 0 | 0 | 523200 | 0 | 0 | 0 |
| Q00613 | 0 | 1021400 | 0 | 0 | 0 | 0 |
| Q13303 | 4322900 | 0 | 0 | 0 | 0 | 0 |
| Q13554 | 0 | 0 | 216220 | 0 | 0 | 0 |
| Q2TBE0 | 1709400 | 0 | 0 | 0 | 0 | 0 |
| Q53T94 | 0 | 1764100 | 0 | 0 | 0 | 0 |
| Q5HYM0 | 0 | 0 | 1021900 | 0 | 0 | 0 |
| Q5JWR5 | 0 | 785220 | 0 | 0 | 0 | 0 |
| Q5SRE5 | 0 | 0 | 1066300 | 0 | 0 | 0 |
| Q5T2E6 | 1219800 | 0 | 0 | 0 | 0 | 0 |
| Q6UUV9 | 0 | 0 | 1685800 | 0 | 0 | 0 |
| Q8N6L1 | 3566900 | 0 | 0 | 0 | 0 | 0 |
| Q8N884 | 0 | 330670 | 0 | 0 | 0 | 0 |
| Q8NDC0 | 0 | 0 | 694080 | 0 | 0 | 0 |
| Q96DM3 | 0 | 2215900 | 0 | 0 | 0 | 0 |
| Q96KQ7 | 33064000 | 0 | 0 | 0 | 0 | 0 |
| Q96L93 | 540340 | 0 | 0 | 0 | 0 | 0 |
| Q96PE3 | 0 | 0 | 4891000 | 0 | 0 | 0 |
| Q9BQB6 | 0 | 0 | 5043100 | 0 | 0 | 0 |
| Q9BTF0 | 2411200 | 0 | 0 | 0 | 0 | 0 |
| Q9BUM1 | 0 | 730740 | 0 | 0 | 0 | 0 |
| Q9NQZ5 | 1273200 | 959090 | 1232500 | 0 | 0 | 0 |
| Q9NRP0 | 17107000 | 0 | 0 | 0 | 0 | 0 |
| Q9NUN5 | 1561100 | 0 | 3657100 | 0 | 0 | 0 |
| Q9NYU1 | 0 | 1204100 | 1647600 | 0 | 0 | 0 |
| A4D256 | 0 | 8118300 | 0 | 0 | 0 | 0 |
| Q96JB8 | 0 | 1404700 | 0 | 0 | 0 | 0 |
| Q9P2D7 | 3272100 | 0 | 0 | 0 | 0 | 0 |

**Table S4.** Tumour volume on day 1 and day 19 as well as the tumour weight, tumour inhibition rate and relative tumour proliferation rate.

| Group | Tumor volume (mm^3^) | | Tumor weight (g) | Inhibition rate (%) | Relative tumour proliferation rate (%) |
| --- | --- | --- | --- | --- | --- |
|  | Day 1 | Day 19 |  |  |  |
| Ctrl | 52.25±21.36 | 3123.15±1003.29 | 2.43±0.90 | 0 | 100 |
| ACT001 | 56.56±16.10 | 789.79±851.11 | 0.65±0.78 | 73.20 | 23.36 |
| Cisplatin | 55.41±20.16 | 977.24±838.02 | 0.80±0.77 | 67.21 | 29.51 |
| ACT001+Cisplatin | 55.05±12.26 | 176.61±177.56 | 0.15±0.17 | 93.88 | 5.56 |

**Supplementary figures**

**
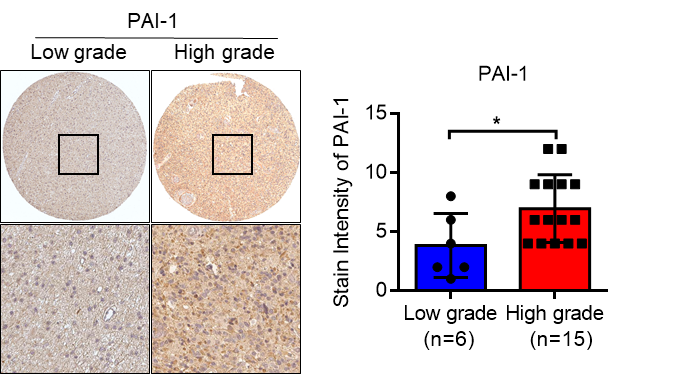
**

**Figure S1.** IHC analysis of PAI-1 expression in low grade and high grade glioma specimens in the human protein atlas datasets. Results showed that the PAI-1 expression gradually increased with the increase of glioma grade.

**
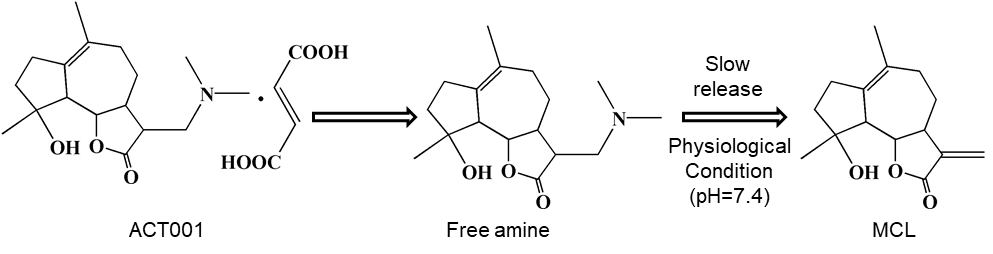
**

**Figure S2.** The relationship between ACT001 and MCL^1^


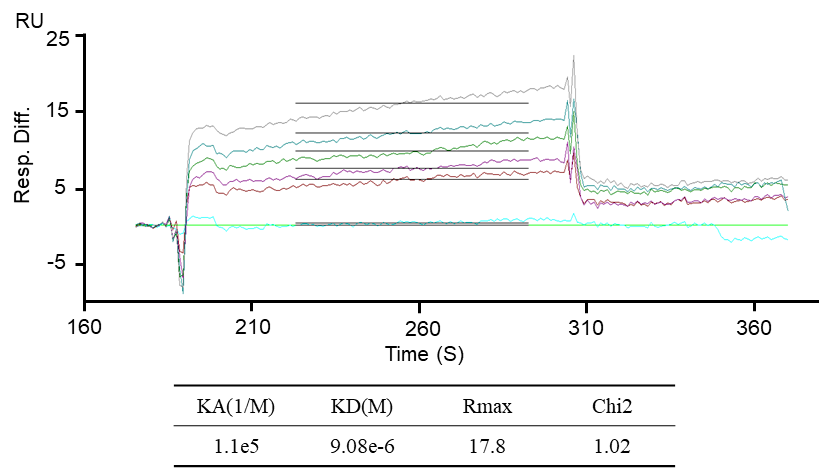


**Figure S3.** After being incubated with different concentrations of ACT001 (0.78125 μM-50 μM) in 1×PBS (pH 7.4) at 37℃ overnight, the solution after incubation was injected into the PAI-1-bound sensor chip.

**References**

1. Li J, Li S, Guo J, Li Q, Long J, Ma C*, et al.* Natural Product Micheliolide (MCL) Irreversibly Activates Pyruvate Kinase M2 and Suppresses Leukemia. *Journal of medicinal chemistry* 2018, **61**(9)**:** 4155-4164.
